# Supplementary material for: iU-ExM: nanoscopy of organelles and tissues with iterative ultrastructure expansion microscopy
Source: Nat Commun. 2023 Nov 30;14:7893. doi: 10.1038/s41467-023-43582-8 (PMC10689735; doi:10.1038/s41467-023-43582-8)
Supplement: Supplementary file 1 — Supplementary_information [file 41467_2023_43582_MOESM1_ESM.pdf]

Supplementary Information

iU-ExM: nanoscopy of organelles and tissues with iterative ultrastructure expansion microscopy

Vincent Louvel[1], Romuald Haase[2], Olivier Mercey[1], Marine. H. Laporte[1], Thibaut Eloy[3], Étienne Baudrier[3], Denis Fortun[3], Dominique Soldati-Favre[2], Virginie Hamel[1]*and Paul Guichard[1]*

Affiliations

[1]Department of Molecular and Cellular Biology, University of Geneva, Geneva, Switzerland.

[2]Department of Microbiology and Molecular medicine, University of Geneva, Geneva, Switzerland.

[3]ICube - UMR7357, CNRS, University of Strasbourg

[*]co-corresponding authors

Correspondence to: virginie.hamel@unige.ch and paul.guichard@unige.ch

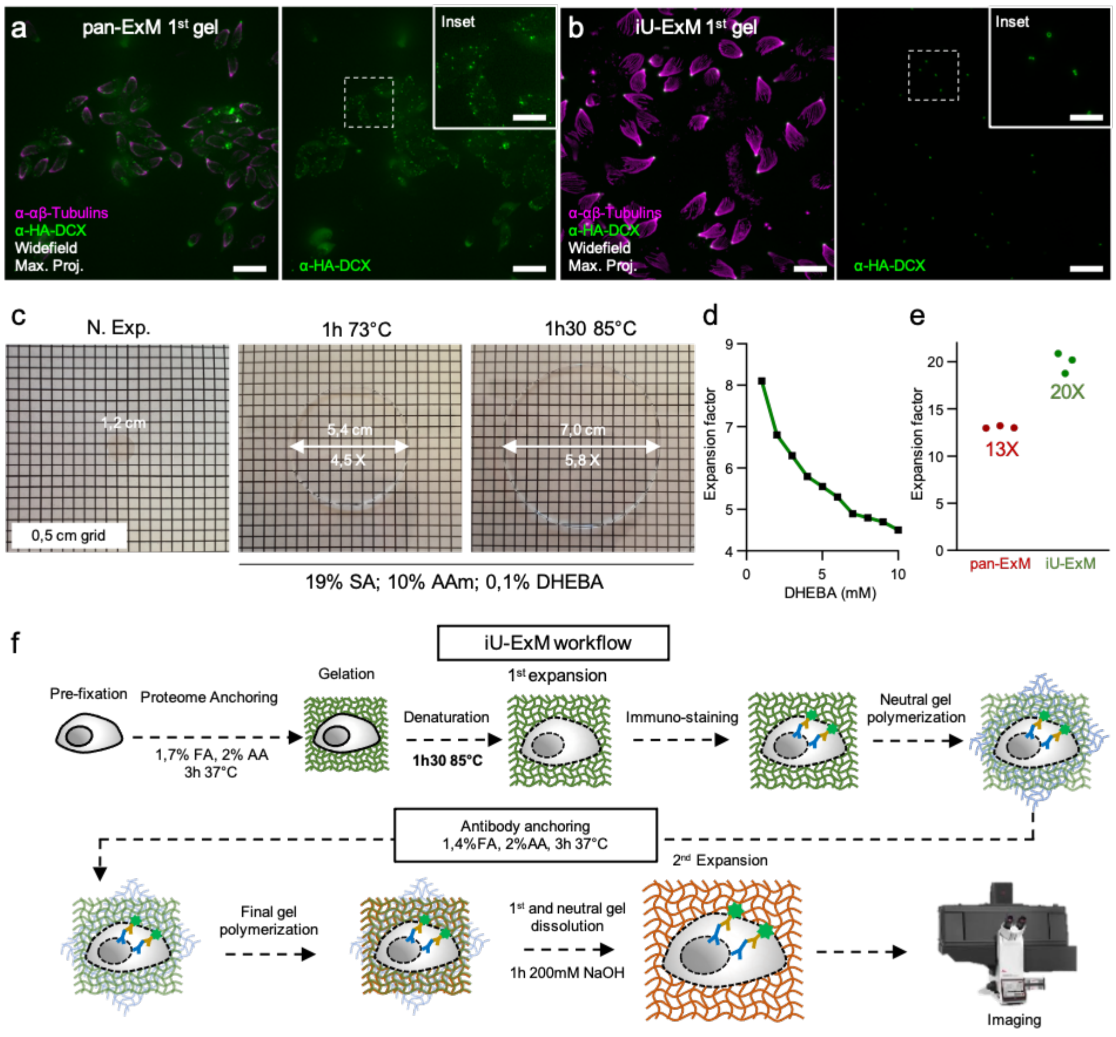

Supplementary Figure 1. iU-ExM workflow and improvement in expansion factor and staining. (**a**) Widefield image of *T. gondii* tachyzoites stained for tubulin and HA-DCX on the 1st expanded gel after 1h denaturation at 73°C. Right panel: HA-DCX channel showing no specific signal at the level of the conoid. Scale bar: 10 µm, 1 µm (inset) non-corrected. (**b**) Widefield image of *T. gondii* tachyzoites stained for tubulin and HA-DCX on the 1st expanded gel after 1h30 denaturation at 85°C. Right panel: HA-DCX channel showing here the characteristic conoid staining of DCX demonstrating the gain in S/N ratio with iU-ExM denaturation conditions. Scale bar: 10 µm, 1 µm (inset) non-corrected (**c**) Pictures of gels highlighting the gain in expansion factors. Left: Non-expanded gel size. Middle: 1st gel after 1h at 73°C denaturation showing 4.5X of gel expansion. Right: 1st gel after 1h30 at 85°C showing 5.8X of gel expansion factor. (**d**) Graph representing the expansion factor in function of the DHEBA concentration. 0.1% DHEBA corresponds to 5 mM. (**e**) Final expansion factor after the 2nd round of expansion based on nuclei cross-section between pan-ExM protocol and iU-ExM highlighting the gain in expansion factor. (**f**) Workflow of the iU-ExM procedure optimised from the pan-ExM protocol. Scale bar: 10 µm non-corrected for the expansion factor. Both (a) and (b) were imaged with the same illumination conditions.

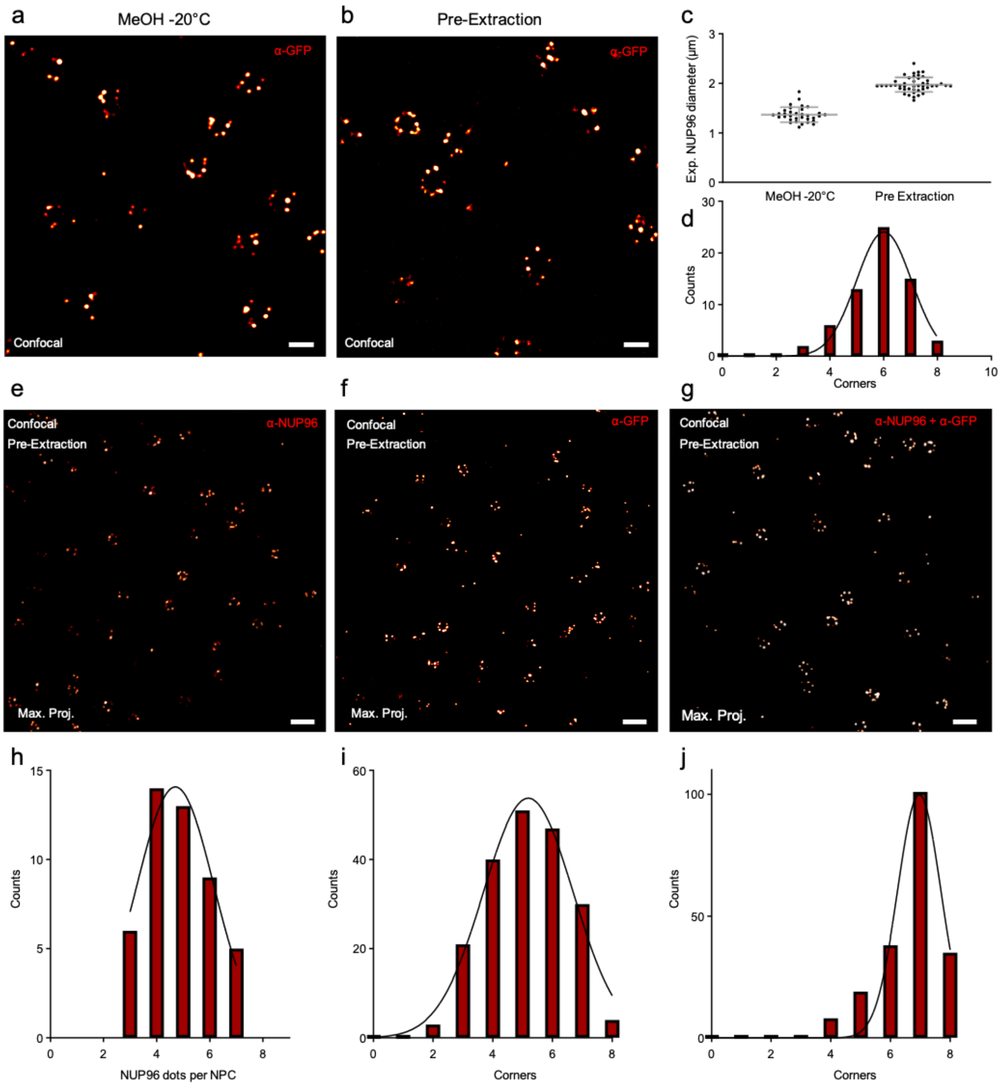

Supplementary Figure 2. Optimizations of nuclear pores fixations and staining. (**a-b**) iU-ExM confocal images of U2OS NUP96-GFP cells fixed with Methanol -20°C (MeOH, b) or the pre-extraction protocol from [13] (a) and stained with α-GFP antibody. Scales bars:1 µm non-corrected (a), 2 µm non-corrected (b). (**c**) Quantification of the expanded diameter of NUP96-GFP cells fixed with methanol fixation or after pre-extraction. Pre-extraction: N= 45 (average +/- standard error = 1.9 +/- 0.15 µm), MeOH: N=33 (average +/- standard error = 1.4 +/- 0.15 µm) both conditions expanded on the same day with the same solutions. Student t-test. ****: p-value <0.0001 (**d**) Counts of the number of NUP96 spots (corners) per NPC under MeOH fixation, N= 64 NPCs from 3 independent experiments. Continuous black line: gaussian regression (R^2= 0.98). (**e, f, g**) iU-ExM confocal images of pre-extracted U2OS NUP96-GFP cells stained with either primary antibody, α-NUP96 (e), α-GFP (f), or a combination of both antibodies (g) Scales bars: 240 nm corrected. (**h, i, j**) Quantification of the number of NUP96 dots (corners) per NPC with either α-NUP96 antibody (h), α-GFP antibody (i), or a combination of both antibodies (j). α-NUP96: N= 47 from one experiment. Continuous black line: gaussian regression (R^2=0.92). α-GFP: N= 196 NPCs from 3 independent experiments. Continuous black line: gaussian regression (R^2= 0.98). MIX: N= 202 NPCs from 3 independent experiments. Black continuous line: gaussian regression (R^2= 0.96). (Note that this graph is identical to Fig. 1f and serves solely for comparison purposes). Continuous black line: gaussian regression (R^2=0.96).

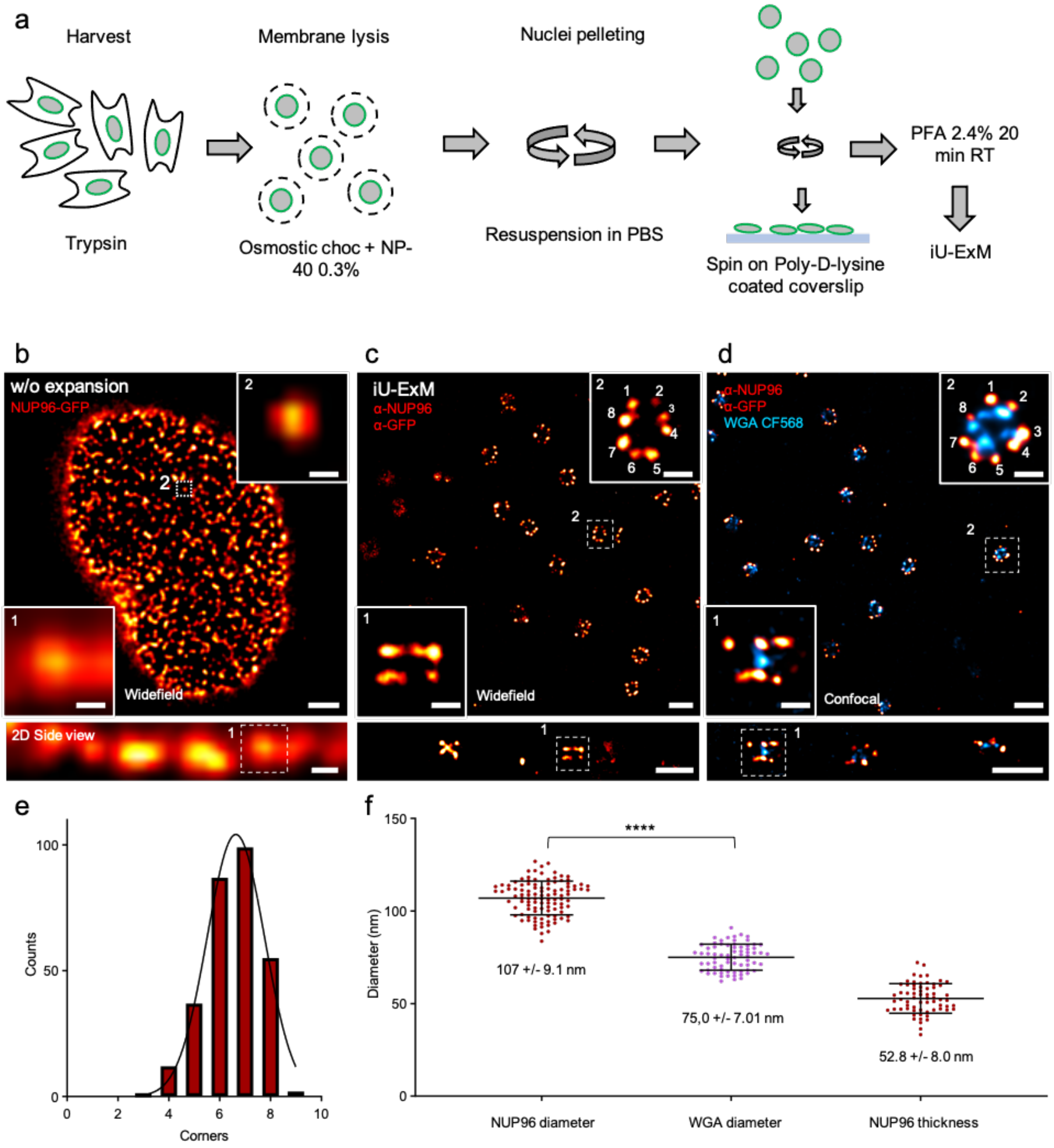

Supplementary Figure 3. iU-ExM on isolated nuclei. (a) Scheme of the nuclei isolation protocol from [50]. **(b)** Widefield image of an isolated nuclei from NUP96-GFP cells (GFP signal, red hot) (100x, 1.47 NA objective). Scale bars: 2 µm (upper image), 500 nm (lower image, side view), 100 nm (insets). **(c)** iU-ExM widefield image of NPCs stained for NUP96 (red-hot, antibody mix anti-GFP and anti-NUP96) (100x, 1,4 NA objective). Scale bars: 200 nm corrected, 50 nm (insets). **(d)** iU-ExM dual color confocal image of NPC stained with WGA-CF568 (cyan) and NUP96 (red hot, antibody mix). Scales bars: 200 nm corrected, 50 nm (insets). **(e)** Distribution of the number of NUP96 positive corners per NPC N= 293 NPC from 3 independent experiments. Red continuous line: gaussian regression (R^2=0.98). **(f)** Quantification of the diameters of the WGA and NUP96 signals and the distance between the nucleoplasmic and cytoplasmic NUP96 rings (NUP96 thickness). Note that the reported NUP96 diameter of 107 nm is used as a proxy to fix the expansion factor. NUP96 diameter: N= 108 (average +/- standard error =107 +/- 9.1 nm), WGA: N= 71 (Average +/- standard error = 75 +/- 7 nm), NUP96 thickness: N= 65 (Average= = 52 +/- 7.9 nm), from 3 independent experiments. ****: P-value < 0,0001, Mann-Whitney test.

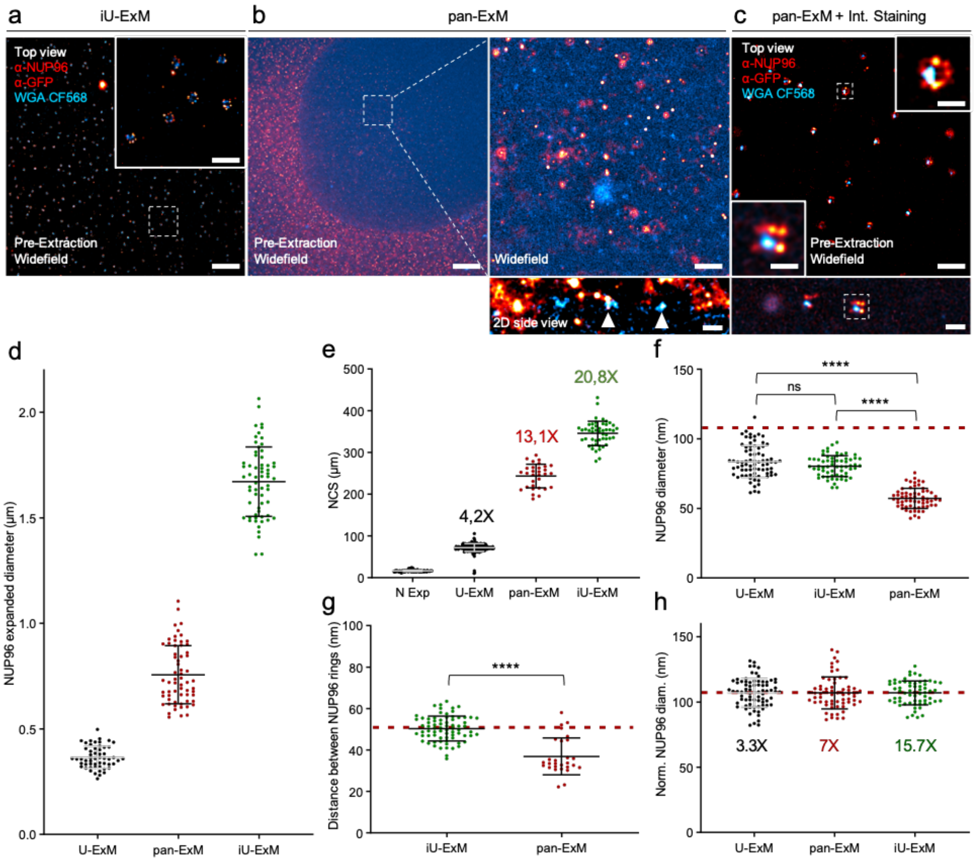

Supplementary Figure 4. iU-ExM compared to U-ExM and pan-ExM. (a-b) Full field of view of Pre-Extracted U2OS NUP96-GFP cells in iU-ExM (**a**) or pan-ExM (**b**). Widefield, Maximum projection. White arrowheads indicate NPCs. Scale bar: 20 µm (**a**- left (**b**)), 5 µm (inset (**a**) and upper right (**b**)), 1 µm (lower right image (**b**)), all non-corrected. (**c**) pan-ExM with intermediate staining. Scale bar: 5 µm (upper image), 1 µm (lower image and insets). (**d**) Measurements of NUP96 diameter in U-ExM, iU-ExM or pan-ExM + intermediate staining. U-ExM: N=55 NPCs (average +/- standard error= 0.4 +/- 0.05 µm), pan-ExM: N=63 NPCs (average +/- standard error=0.8 +/- 0.1 µm), iU-ExM: N=63 NPCs (average +/- standard error=1.7 +/- 0.2 µm). (**e**) Measurement of the NCS of either Non-expanded (N Exp); U-ExM; pan-ExM; iU-ExM Pre-Extracted U2OS cells. N Exp: N= 384 nuclei (average +/- standard error= 16.8 +/- 1.8 µm), U-ExM: N=290 nuclei (average +/- standard error= 72.1 +/- 9.3 µm); pan-ExM: N= 31 nuclei (average +/- standard error= 243.7 +/- 28.5 µm); iU-ExM: N=49 nuclei (average +/- standard error= 349.5 +/- 29.1 µm). (**f**) Quantification of the NUP96 diameter corrected by the average nuclei expansion factor with either U-ExM, iU-ExM or pan-ExM. U-ExM: N= 77 NPCs (average +/- standard error= 83.6 +/- 11.5 nm), iU-ExM: N= 63 NPCs (average +/- standard error= 80.4 +/- 7.4 nm), pan-ExM: N= 63 NPCs (average +/- standard error= 57.2 +/- 7.2 nm). (**g**) Quantification of the nuclear pores thickness corrected with nuclei expansion factor. iU-ExM: N= 73 NPCs (average +/- standard error= 50.35 +/- 6.0 nm), pan-ExM: N= 28 (average +/- standard error= 36.9 +/- 8.9 nm) from 2 independent experiments. (**h**) Expansion factor calculation using 107 nm as a reference with either U-ExM, iU-ExM or pan-ExM. U-ExM: N= 77 NPCs (average +/-standard error= 107.0 +/- 11.0 nm), pan-ExM: N= 63 NPCs (average +/-standard error= 107.0 +/- 12.3 nm), iU-ExM N= 63 NPCs (average +/- standard error= 107.0 +/- 9.1 nm). One way ANOVA (f). Kruskal Wallis test (g). ****: p-value<0.0001. In (**f**) and (**h**), the red dashed line represents the published values for NUP96 diameter of 107 nm and in (**g**) the thickness.

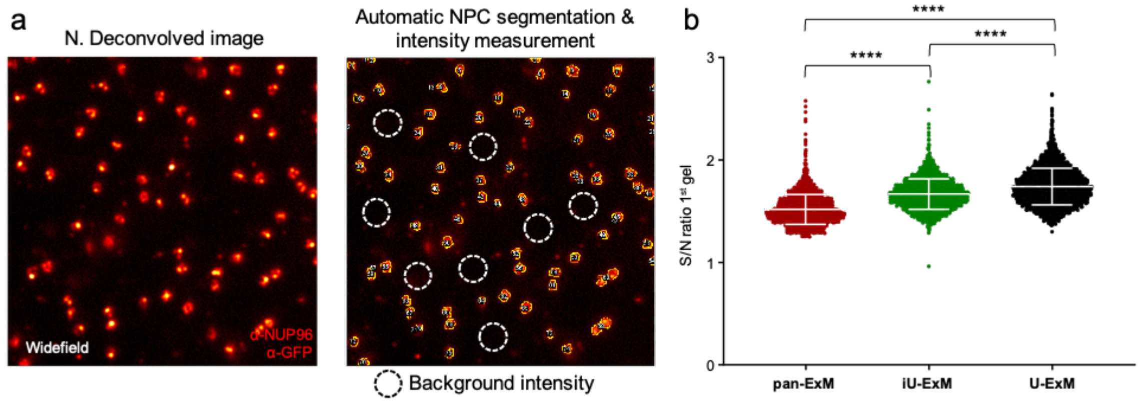

Supplementary Figure 5. NPCs Signal-to-noise ratio with iU-ExM, pan-ExM and U-ExM: (**a**) Quantification of NPCs S/N ratio. Left, widefield picture of NPCs expanded with U-ExM and stained with the NUP96 antibody mix. Right, NPC are automatically segmented and two intensity signals are measured, the NPCs signal and the background. (**b**) Quantification of the NPCs S/N ratio. pan-ExM: N= 3600 NPCs (average +/- standard error= 1.5 +/- 0.1), iU-ExM: N= 3053 NPCs (average +/- standard error= 1.7 +/- 0.1), U-ExM: N= 5003 NPCs (average +/- standard error= 1.7 +/- 0.2) from 3 independent experiments. Note that the intensity has not been corrected with the expansion factor. Kruskal Wallis test. ****: p-value<0.0001.

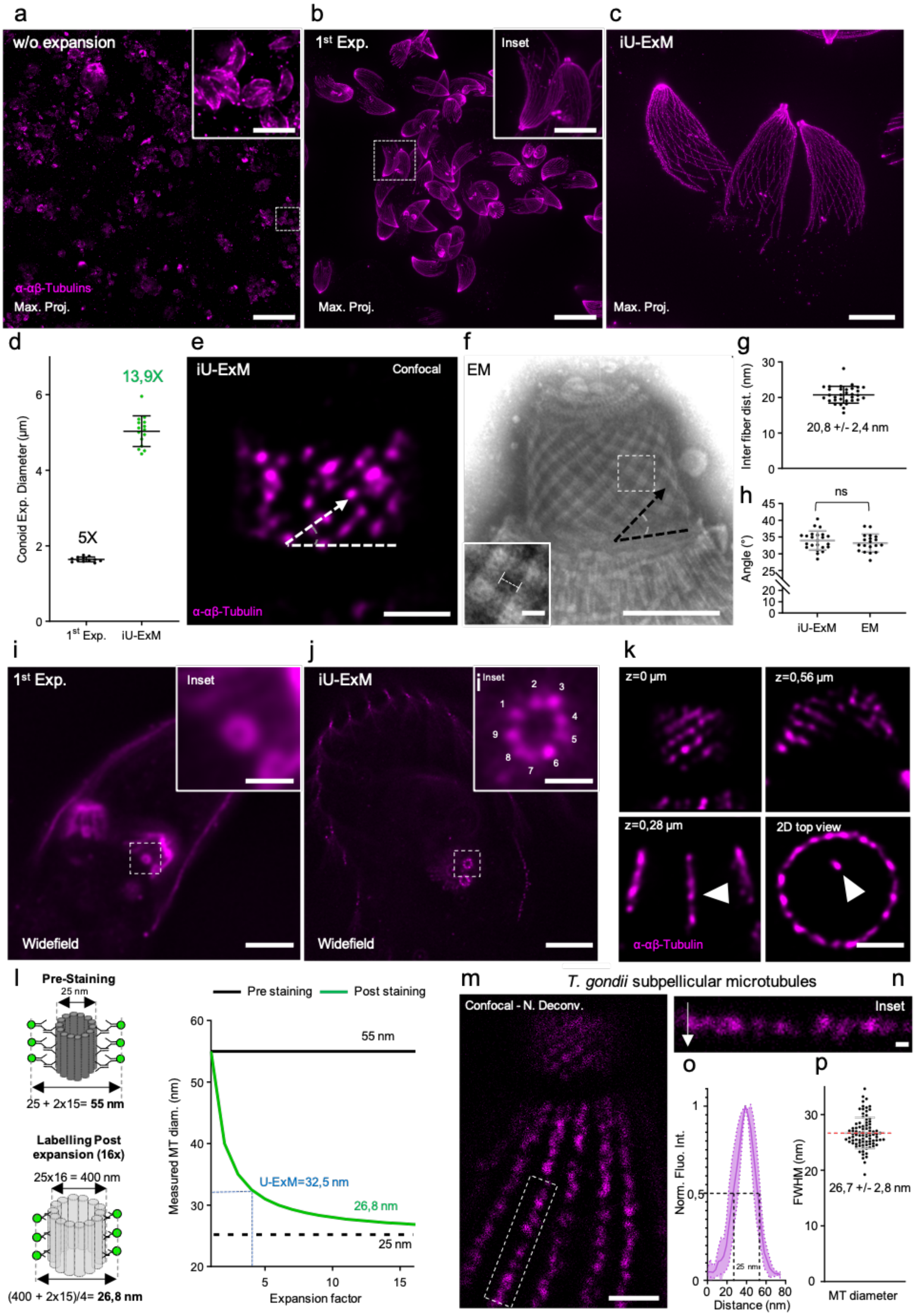

Supplementary Figure 6. iU-ExM on *Toxoplasma gondii*. (a-c) Full field of view of non-expanded (a), expanded once (b) or iU-ExM expanded (c) *T. gondii* tachyzoites stained for tubulin (magenta). Scale bars (a-c): 50 µm, 10 µm (inset) non-corrected.**(d)** Quantification of the conoid diameter at its proximal side. The expansion factor is calculated by dividing the average diameter value by 380 nm. 1st Expansion (1st Exp.): N= 12 conoids (average +/- standard error= 1.6 +/- 0.1 µm) iU-ExM: N= 15 conoids (average +/- standard error= 5.0 +/-

0.4 µm), from the same consecutive expansion. (**e-f**) iU-ExM image of a conoid with dashed lines indicating how angles were measured. Scale bar: 200 nm, corrected from the conoid's expansion factor. (**f**) EM image of a conoid. Inset: inter-fibers distance. Scale bar: 200 nm, 20 nm (inset). (**g**) Measurement of the inter-fibers distance from EM images. N= 32 (average +/- standard deviation= 20.8 +/- 2.4 nm) from one experiment. (**h**) Quantification of the angles measured in (**e**), iU-ExM) and (**f**), EM. iU-ExM: N=23 fibers (average +/- standard error= 33.9 +/- 2.8 °), EM: N=19 fibers (average +/- standard error=33.2 +/- 2.6°) from one experiment. Unpaired student t-test, ****: p-value<0.001. (**i-j)** Representative images of single (i) or iU-ExM (j) expanded *T. gondii* revealing the 9-fold organization of the centriolar microtubule wall. Scale bars: (i-j): 4 µm, 500 nm (inset) All non-corrected. (**k**) iU-ExM confocal images of conoids at different z-step and one top view showing the intra conoidal microtubules (white arrowheads). Scale bars: 80 nm corrected. (**l**) Graph representing the theoritical diameter of a microtubule with either pr-e or post-staining. (**m**) Raw confocal image of cortical microtubules of *T. gondii* tachyzoite. Scale bar: 80 nm corrected. (**n**) Inset of the dashed box in (**m**), the white arrow represents the direction of the plot profiles done for measuring the FWHM (**o**). Scale bar: 25 nm corrected. (**p**) Quantification of the FWHM of the microtubules, N= 86 microtubules (average +/- standard error= 26.7 +/- 2.8 nm). Except specified, all experiments were performed at least 3 times.

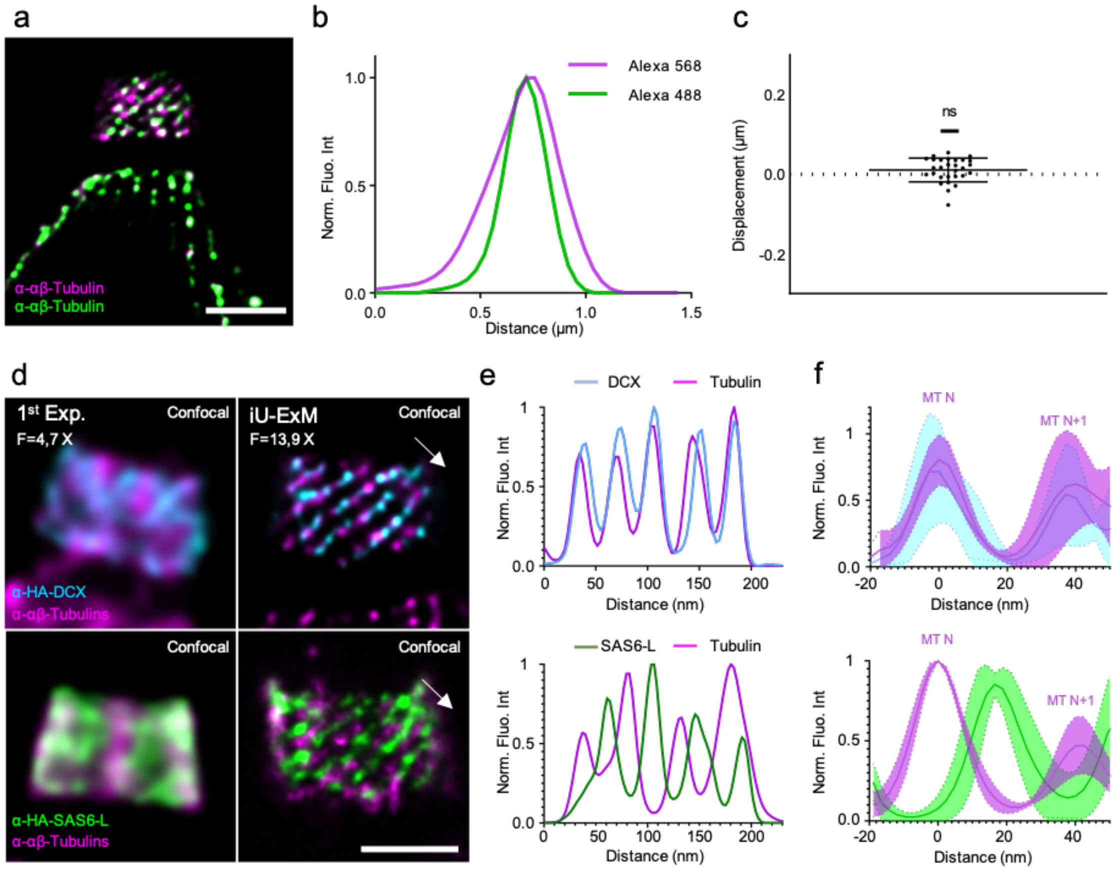

Supplementary Figure 7. Dual color iU-ExM on *Toxoplasma gondii* conoid. (**a**) iU-ExM confocal image of a *T. gondii* tachyzoite conoid stained with primary antibodies against tubulin and two secondaries with different colors (Alexa 488 and rabbit Alexa 568) to test potential color shift. Scale bar: 5 µm non-corrected. (**b**) Plot profile of a tubulin fiber marked with either Alexa 488 or Alexa 568. (**c**) Quantification of the displacement between Alexa 488 and Alexa 568 peaks. N= 29 tubulin fibers (average +/- standard error= 0.01 +/- 0.03 µm) from 2 independent experiments. One sample student t-test with comparison to 0: non significative. (**d**) Widefield images of single (left column) or iU-ExM (right column) expanded *T. gondii* conoids stained for HA-tagged DCX (cyan) or HA-tagged SAS6-L (green). and tubulin (magenta). Scale bar: 200 nm corrected. (**e**) Plot profiles (white arrow in d) through the conoid expanded with iU-ExM showing the position of DCX and SAS6-L relative to the tubulin fibers. (**f**) Pool of 6 plot profiles from different images. MT N: tubulin fibers at one given position; MT N+1: neighboring tubulin fibers.

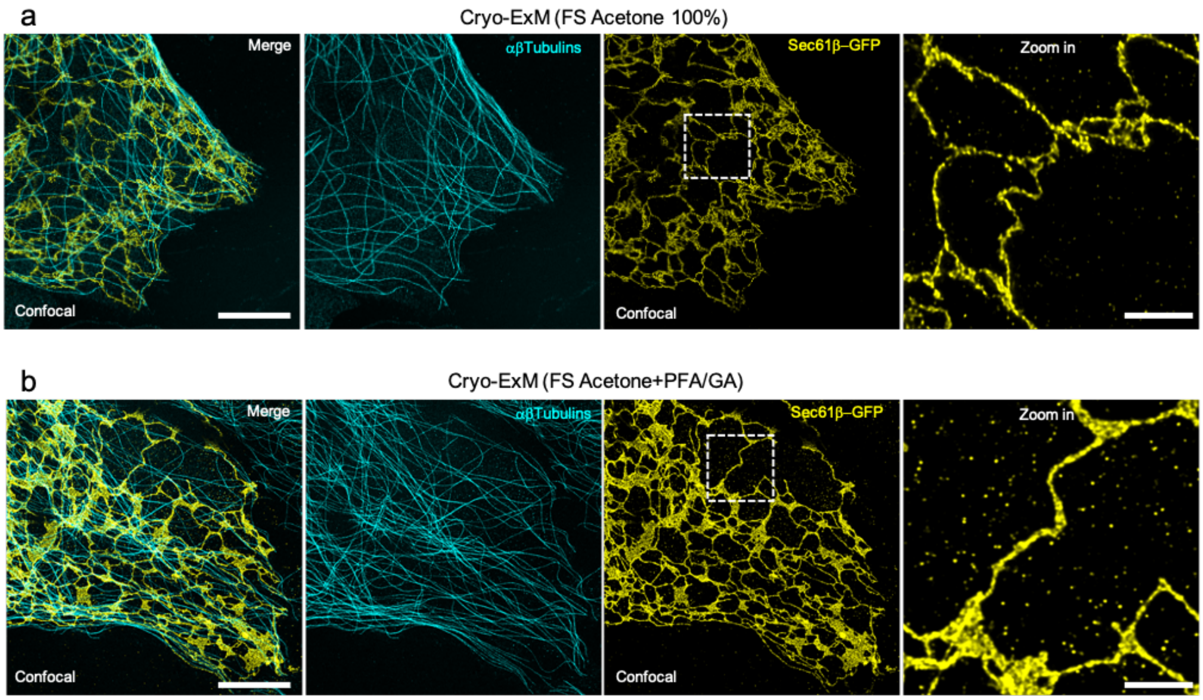

Supplementary Figure 8. **Endoplasmic reticulum membrane preservation in Cryo-ExM.** **(a)** Confocal image of an expanded (U-ExM) Sec61β-GFP transfected U2OS cell after cryo-fixation and freeze substitution (FS) in acetone 100%. Cells were stained for anti-GFP (yellow) and anti-tubulin (cyan) antibodies labelling the endoplasmic reticulum (Sec61β-GFP) and the microtubules respectively. The white dashed square indicates the inset (zoom in) showing a slightly collapsed endoplasmic reticulum in 100% acetone FS conditions. Scale bars: 5 mm and 1 mm (inset) non-corrected. **(b)** Confocal image of an expanded U2OS after cryo-fixation and freeze substitution (FS) in acetone supplemented with PFA 0.5% and GA 0.02%. Cells were stained for anti-GFP (yellow) and anti-tubulins (cyan) antibodies. The white dashed square indicates the inset (zoom in) showing a non-collapsed endoplasmic reticulum in PFA/GA FS conditions. Scale bars: 5 mm and 1 mm (inset) non-corrected.

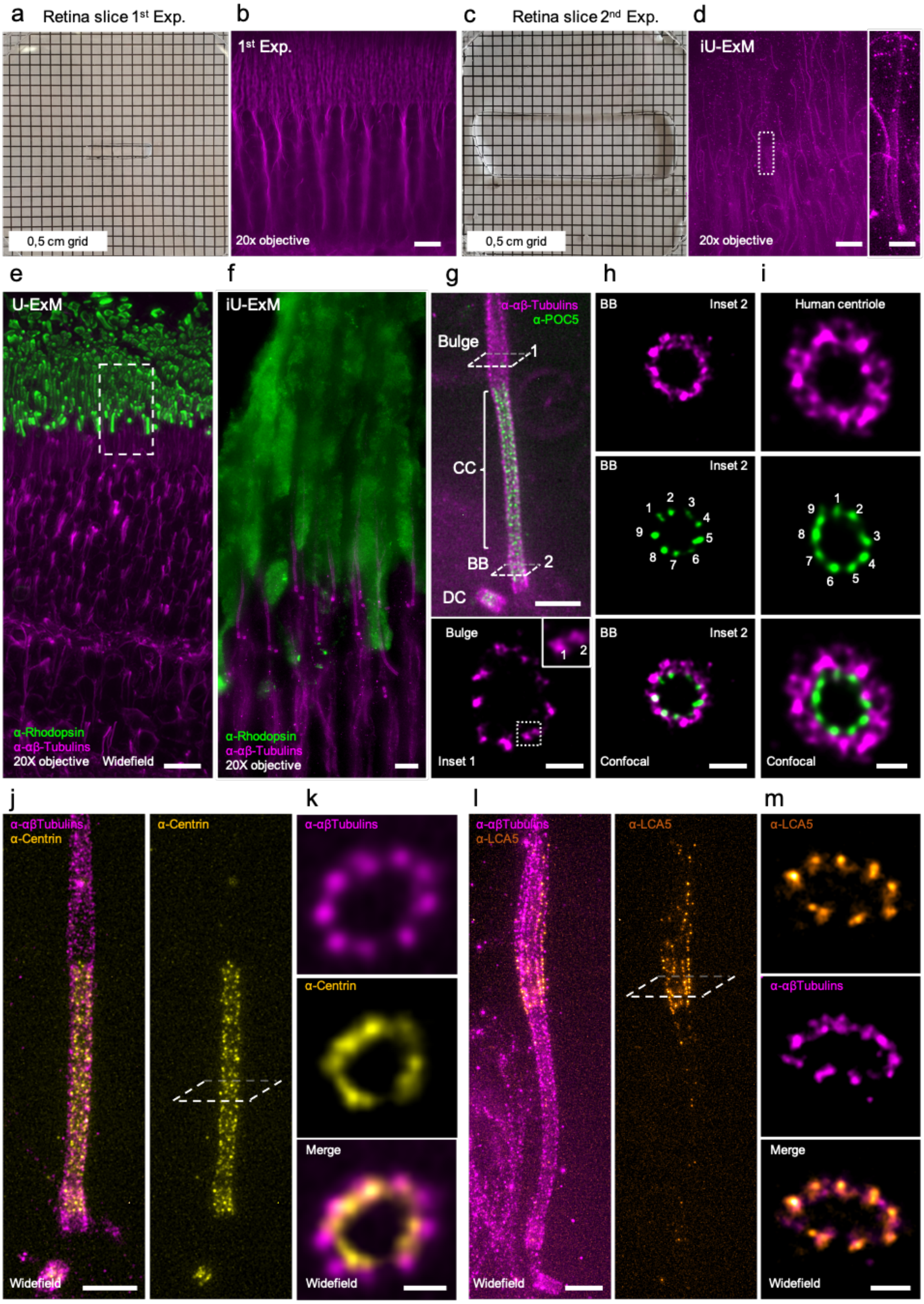

Supplementary Figure 9. iU-ExM on retinal tissue. (**a, c**) Gel of a retina slice after the first (**a**) and second (**b**) expansion, deposited on a 0.5*0.5 cm grid. (**b, d**) Full field of view of the retina slice stained for tubulin in widefield microscopy after the first expansion (**b**) and second expansion(**d**), revealing the connecting cilium (inset). Scale bars: 50 µm non-corrected, 10 µm non corrected (inset). **(e)** U-ExM on an expanded mouse retinal tissue. Scale bar: 10 µm non-

corrected. **(f)** iU-ExM on an expanded mouse retinal highlighting the gain in expansion and resolution compared to U-ExM. Scale bar: 10 µm non-corrected. **(g)** Top: widefield image of an iU-ExM expanded photoreceptor cell. BB: Basal Body, CC: connecting cilium, DC: Daughter Centriole. Scale bar: 500 nm corrected. Inset 1: confocal top view of the bulge region. Note that individual microtubules from the microtubule doublets are detectable. Scale bar: 500 nm corrected. (**h)** Top view of a basal body underlying the CC of an iU-ExM expanded photoreceptor cell unveiling the 9-fold organization of POC5 (green). Note that the tubulin (magenta) signal at the level of BB is less clear than that at the CC (Fig. 5d). Scale bar: 200 nm corrected. **(i)** Top view of a human centriole in iU-ExM showing the conservation of the 9-fold pattern of POC5 (green) in human centrioles. Note also that similarly to the BB, the tubulin (magenta) signal is not nicely resolved at centrioles in human cells. Scale bar: 50 nm corrected. **(j)** iU-ExM on a connecting cilium stained for tubulin (magenta) and Centrin (yellow). Scale bar: 400 nm corrected. (**k**) Cross section of the connecting cilium with tubulin and Centrin staining. Scale bar: 100 nm corrected. (**l**) iU-ExM widefield images of a connecting cilium stained for tubulin (magenta) and LCA5 (orange). Scale bar: 400 nm corrected. (**m**) Cross section of the bulge region with tubulin and LCA5 staining. Scale bar: 100 nm corrected.

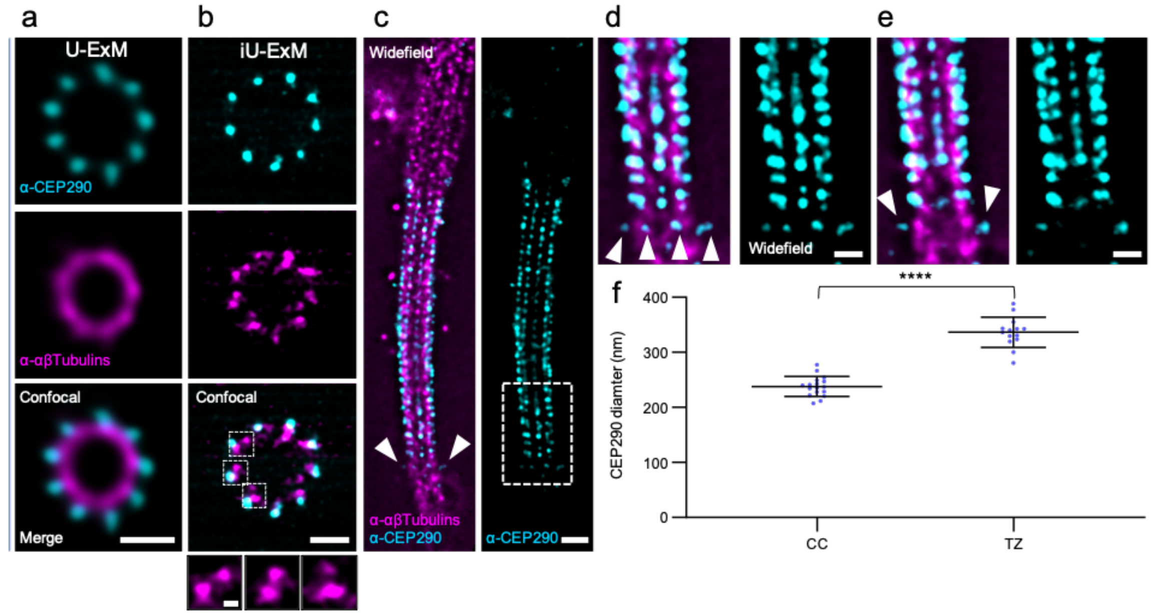

Supplementary Figure 10. CEP290 localization in photoreceptors

(a) Top view of a CC stained for tubulin (magenta) and CEP290 (cyan) in U-ExM. Scale bar: 200 nm, corrected with gel expansion factor. (**b**) Top view of a CC stained for tubulin and CEP290 in iU-ExM. Scale bar: 100 nm, corrected with the basal body proximal diameter of 225 nm. Bottom part: inset of the area marked with dashed boxes showing microtubule doublets. (**c**) iU-ExM on a CC stained with tubulin and CEP290. Scale bar: 200 nm corrected. (**d**) Close up on the transition zone marked with the rectangular dashed box in (c). The image corresponds to a Z slice at the surface of the CC showing the external CEP290 signals at the level of the transition zone (white arrowheads). Scale bar: 100 nm corrected. (**e**) Image of Z slice towards the centre of the CC, showing the external CEP290 signal at the level of the transition zone (white arrowhead). Scale bar: 50 nm corrected. (**f**) Quantification of the CEP290 signal diameter at the transition zone and at the level of the CC. CC: N=17 connecting cilium (average +/- standard error= 237.9 +/- 18.2 nm). TZ: N= 14 connecting cilium (average +/- standard error=336.5 +/- 27.3 nm) from 2 independent experiments. ****: p-value<0.001. Unpaired student t-test.

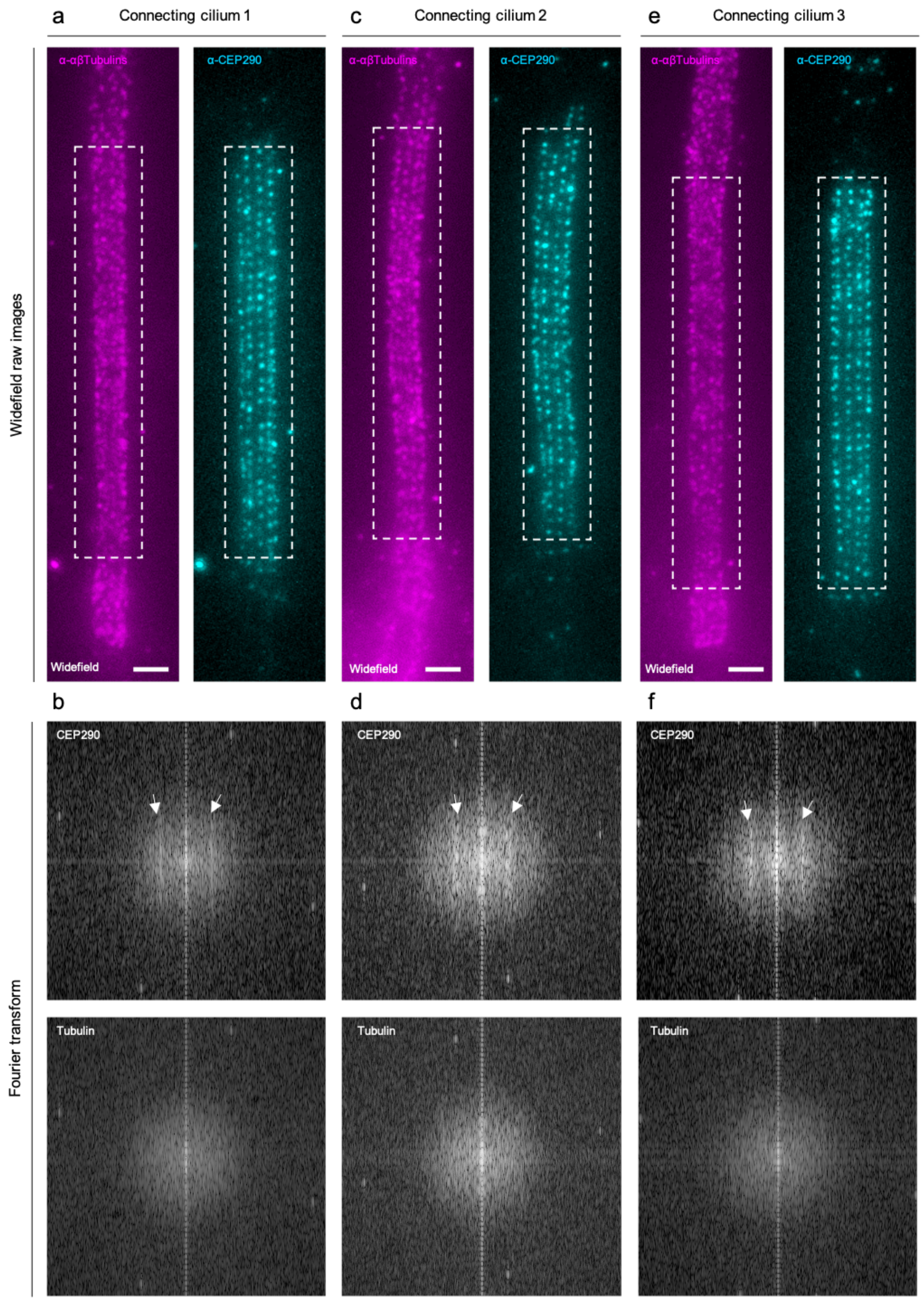

Supplementary Figure 11. Structural periodicity of CEP290 along the connecting cilium (**a, c, e)** Three examples of iU-ExM applied on the photoreceptor connecting cilium with tubulin (magenta, left) and CEP290 (cyan, right). The rectangular dashed box marks the region from which the Fourier transform was calculated. Scale bar: 200 nm corrected. (**b, d, f**) Fourier transform for the area marked in the (a, c, e) panels demonstrating the CEP290 signal periodicity (white arrows) along the connecting cilium. Note that the periodicity of the microtubule blades is not detectable here.

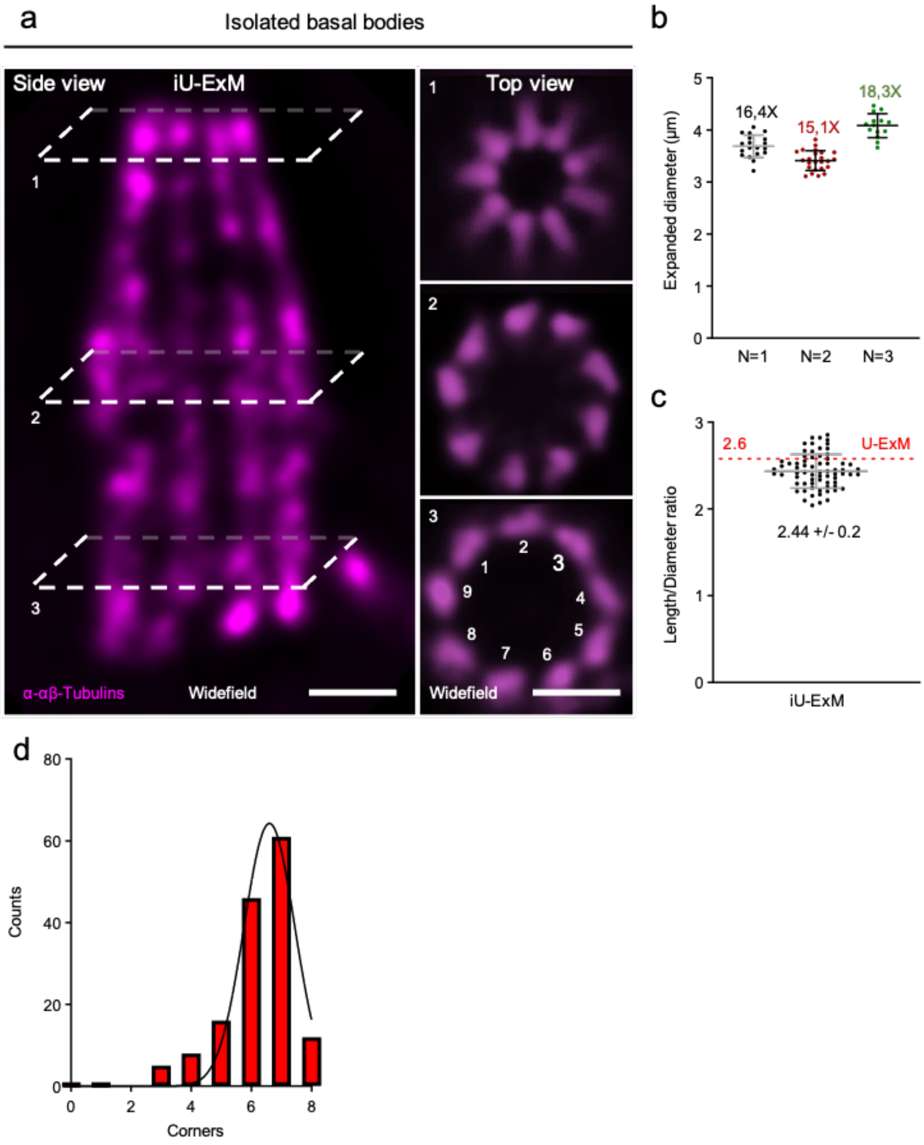

Supplementary Figure 12. iU-ExM on *Chlamydomonas reinhardtii* isolated basal bodies and nuclear pores (a) Isolated basal body (BB) from *C. reinhardtii* expanded with iU-ExM and stained with tubulin (magenta). Left image, BB from side view with microtubule blades seen longitudinally. Right images: BB top views corresponding to the indicated z-position from the dashed boxes on the left image. Scales bars: 100 nm, corrected. **(b)** Quantification of the diameter of the proximal area of the basal body for 3 independent experiments. The average value is divided by 225 nm (centriole diameter[51]) to calculate the expansion factor. N=1 (N=19 centrioles, average +/- standard error= 3.7 +/- 0.2 µm), N=2 (N=24 centrioles, Average +/- standard error= 3.4 +/- 0.2 µm), N=3 (N= 14 centrioles, average +/- standard error= 4.1 +/- 0.2 µm). (**c**) Length over diameter ratio, the red line shows the value obtained in U-ExM [6]. N= 69 (Average +/- standard error = 2.4 +/- 0.2) centrioles from 3 independent experiments. (**d**) Quantification of the distribution of the number of NUP96 signal (corners) per NPC. N=149 NPCs from 3 independent experiments.

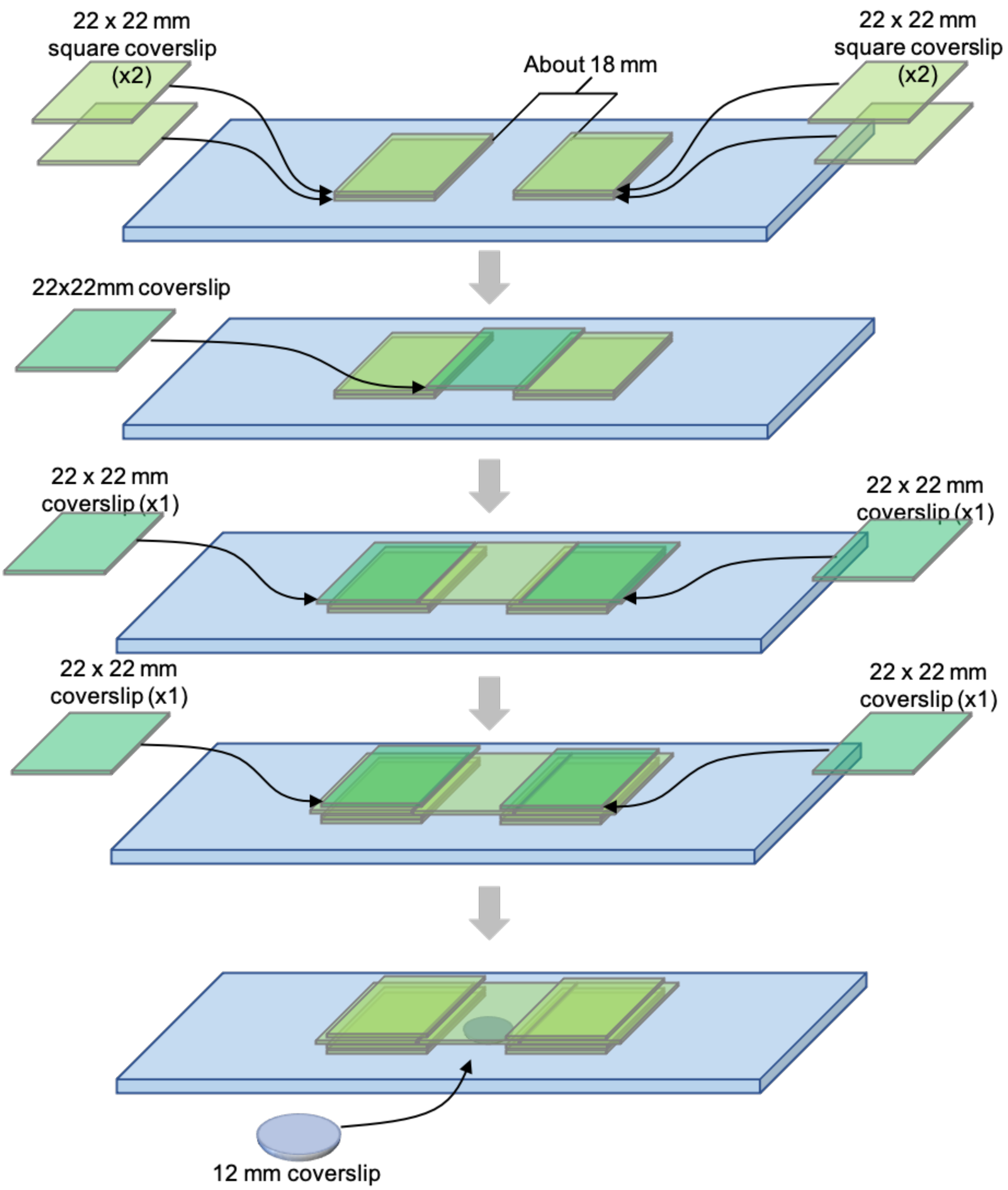

Supplementary Figure 13. **Gelation chamber**
Scheme for building a gelation chamber for samples deposited on 12mm coverslips. The chamber can be built by stacking 22x22mm glass coverslips on a glass slide
