# Supplementary material for: iU-ExM: nanoscopy of organelles and tissues with iterative ultrastructure expansion microscopy
Source: Nat Commun. 2023 Nov 30;14:7893. doi: 10.1038/s41467-023-43582-8 (PMC10689735; doi:10.1038/s41467-023-43582-8)
Supplement: Supplementary file 3 — Description of Additional Supplementary Files [file 41467_2023_43582_MOESM3_ESM.pdf]

Description of Additional Supplementary Files

File Name: Supplementary Movie 1
Description: Widefield movie of top view NPCs from iU-ExM expanded purified NUP96-GFP (red hot) nuclei. The sample was stained with α-GFP and α-NUP96 antibodies and imaged at 100X with a 1.4NA objective. Scale bar: 1 µm corrected.

File Name: Supplementary Movie 2
Description: Widefield movie of NPCs in side view from iU-ExM expanded purified NUP96-GFP (red hot) nuclei. The sample was stained with α-GFP and α-NUP96 antibodies and imaged at 100X with a 1.4NA objective. Scale bar: 1 µm corrected.
