# Supplementary material for: iU-ExM: nanoscopy of organelles and tissues with iterative ultrastructure expansion microscopy
Source: Nat Commun. 2023 Nov 30;14:7893. doi: 10.1038/s41467-023-43582-8 (PMC10689735; doi:10.1038/s41467-023-43582-8)
Supplement: Supplementary file 6 — Reporting Summary [file 41467_2023_43582_MOESM6_ESM.pdf]

nature portfolio

Corresponding author(s): Paul Guichard

Last updated by author(s): Oct 10, 2023

Reporting Summary

Nature Portfolio wishes to improve the reproducibility of the work that we publish. This form provides structure for consistency and transparency in reporting. For further information on Nature Portfolio policies, see our Editorial Policies and the Editorial Policy Checklist.

Statistics

For all statistical analyses, confirm that the following items are present in the figure legend, table legend, main text, or Methods section.

n/a	Confirmed	
☐	☒	The exact sample size (n) for each experimental group/condition, given as a discrete number and unit of measurement
☐	☒	A statement on whether measurements were taken from distinct samples or whether the same sample was measured repeatedly
☐	☒	The statistical test(s) used AND whether they are one- or two-sided<br>*Only common tests should be described solely by name; describe more complex techniques in the Methods section.*
☒	☐	A description of all covariates tested
☒	☐	A description of any assumptions or corrections, such as tests of normality and adjustment for multiple comparisons
☐	☒	A full description of the statistical parameters including central tendency (e.g. means) or other basic estimates (e.g. regression coefficient) AND variation (e.g. standard deviation) or associated estimates of uncertainty (e.g. confidence intervals)
☐	☒	For null hypothesis testing, the test statistic (e.g. F, t, r) with confidence intervals, effect sizes, degrees of freedom and P value noted<br>*Give P values as exact values whenever suitable.*
☒	☐	For Bayesian analysis, information on the choice of priors and Markov chain Monte Carlo settings
☒	☐	For hierarchical and complex designs, identification of the appropriate level for tests and full reporting of outcomes
☒	☐	Estimates of effect sizes (e.g. Cohen's d, Pearson's r), indicating how they were calculated

Our web collection on statistics for biologists contains articles on many of the points above.

Software and code

Policy information about availability of computer code

Data collection	Image acquisition was performed on inverted confocal microscopes Leica TCS SP8 or Stellaris 8, or widefield microscope Leica Thunder DMI8, using a 63x 1.4 NA/100X 1.47 NA oil objective with Lightning or thunder SVCC (small volume computational clearing) mode at max resolution, adaptive as "strategy" and water as Mounting medium to generate deconvolve images. 3D stacks were acquired with 120nm z-interval and an x, y pixel size of 35nm (Leica TCS SP8 and Stellaris 8, or 210nm Z-intervals and an x,y,pixel of 100nm (Thunder DMi8))<br>The code for quantifying the number of corners in nuclear pores is available on github: https://github.com/thibaut1998e/NPC_symmetry_quantification/tree/main
Data analysis	The images were generated with the software ImageJ and graphpad was used for graphical representation and statistics.

For manuscripts utilizing custom algorithms or software that are central to the research but not yet described in published literature, software must be made available to editors and reviewers. We strongly encourage code deposition in a community repository (e.g. GitHub). See the Nature Portfolio guidelines for submitting code & software for further information.

Data

Policy information about availability of data

All manuscripts must include a data availability statement. This statement should provide the following information, where applicable:

- Accession codes, unique identifiers, or web links for publicly available datasets
- A description of any restrictions on data availability
- For clinical datasets or third party data, please ensure that the statement adheres to our policy

The data that support the findings of this study are available as "source data" provided with the manuscript. Further request can be sent to the corresponding authors

Research involving human participants, their data, or biological material

Policy information about studies with human participants or human data. See also policy information about sex, gender (identity/presentation), and sexual orientation and race, ethnicity and racism.

Reporting on sex and gender	N/A
Reporting on race, ethnicity, or other socially relevant groupings	N/A
Population characteristics	N/A
Recruitment	N/A
Ethics oversight	N/A

Note that full information on the approval of the study protocol must also be provided in the manuscript.

Field-specific reporting

Please select the one below that is the best fit for your research. If you are not sure, read the appropriate sections before making your selection.

- [x] Life sciences
- [ ] Behavioural & social sciences
- [ ] Ecological, evolutionary & environmental sciences

For a reference copy of the document with all sections, see nature.com/documents/nr-reporting-summary-flat.pdf

Life sciences study design

All studies must disclose on these points even when the disclosure is negative.

Sample size	We did not use a predetermined sample size. Instead, we captured as many images as possible in each experiment. To ensure statistical confidence, we obtained a minimum of 10 biological replicates for each independent experiment. With the exception of a few specific experiments mentioned in the manuscript, we conducted each experiment three times independently and pooled the data from the three replicates for the final representation. Specific quantification numbers are provided in the figure legend.
Data exclusions	No exclusion was done.
Replication	All experiments were performed at least 3 times except in very few cases (specified in the manuscript). Some replications for isolated Nuclei for Nuclear Pores Complexes analysis were not successful and excluded because of a weak antibody staining.
Randomization	This is not relevant to this study as we selected representative images for every experiments. We did not have any experiments were randomization would be pertinent.
Blinding	This not relevant for our study as specified above.

Reporting for specific materials, systems and methods

We require information from authors about some types of materials, experimental systems and methods used in many studies. Here, indicate whether each material, system or method listed is relevant to your study. If you are not sure if a list item applies to your research, read the appropriate section before selecting a response.

Materials & experimental systems

n/a	Involved in the study	
	☒	Antibodies
	☒	Eukaryotic cell lines
☒		Palaeontology and archaeology
	☒	Animals and other organisms
☒		Clinical data
☒		Dual use research of concern
☒		Plants

Methods

n/a	Involved in the study	
☒		ChIP-seq
☒		Flow cytometry
☒		MRI-based neuroimaging

Antibodies

Antibodies used

- Tubulin monobodies AA344 and AA345
- Rabbit polyclonal anti-POC5 (Bethyl-A303-341A)
- Rabbit polyclonal anti-GFP (TP401, Torrey pines).
- Rabbit polyclonal anti-NUP98-96 (ProteinTech-12329-1-AP).
- Rabbit polyclonal anti-NUP205 (ProteinTech, 24439-1-AP)
- Rat anti-HA (Roche, 11 867 423 001).
- Mouse anti-Rhodopsin (Thermoscientific, MA5-11741).
- Mouse anti-Centrin (Millipore - 04-1624)
- Rabbit anti-LCA5 (Proteintech-19333-1-AP)
- Rabbit anti-CEP290 (Proteintech-22490-1-AP)

The following secondary antibodies were used:
- Goat anti-Rabbit Alexa Fluor 488 IgG H+L (Invitrogen, A11008)
- Goat anti-mouse Alexa 568 IgG H+L (Invitrogen, A11004).
- Goat anti-mouse Alexa 488 IgG H+L (Invitrogen, A11029).
- Goat anti-Guinea Pig 568 IgG H+L (Invitrogen, A11075).
- Goat anti-rat AlexaFLuor 488 IgG H+L (Invitrogen : A21208)

Validation

- Validation AA344: https://oap.unige.ch/journals/abrep/article/view/108
- Validation AA345: https://oap.unige.ch/journals/abrep/article/view/260 and https://oap.unige.ch/journals/abrep/article/view/108
- Validation A303-341A: https://www.biomol.com/products/antibodies/primary-antibodies/general/anti-poc5-a303-341a-tjournal.pbio.3001649
- Validation TP401: western blot: https://www.amsbio.com/rabbit-anti-gfp-pab-tp401
- Validation 12329-1-AP: western blot: https://www.ptglab.com/products/NUP98-Antibody-12329-1-AP.htm
- Validation 24439-1-AP: western blot: https://www.ptglab.com/products/NUP205-Antibody-24439-1-AP.htm
- Validation 11 867 423 001: https://www.sigmaaldrich.com/deepweb/assets/sigmaaldrich/product/documents/248/175/roahahabul.pdf
- Validation MA5-11741: https://www.thermofisher.com/antibody/product/Rhodopsin-Antibody-clone-RET-P1-Monoclonal/MA5-11741.
-Validation 22490-1-AP: https://www.ptglab.com/products/CEP290-Antibody-22490-1-AP.htm
- Validation 1933-1-AP: https://www.ptglab.com/products/LCA5-Antibody-19333-1-AP.htm
- Validation 04-1624: https://www.sigmaaldrich.com/CH/de/product/mm/041624

Eukaryotic cell lines

Policy information about cell lines and Sex and Gender in Research

Cell line source(s)

U2OS-NUP96-GFP: Gift from Jonas Ries Lab (unknown supplier, same cell line than in this paper: https://www.nature.com/articles/s41592-019-0574-9). Non transformed U2OS: ATCC-HTB-96. Toxoplasma Parasites are cultured and amplified in Human Foreskin Fibroblasts (HFF, ATCC, CRL1634). T. gondii cell line (RHΔhxgprtΔKu80 - Tir1 (Parental line for all -mAiD-HA strains generated in this study)): Tir1 expressing line (generation of mAID-HA strains) was provided by Pr. Sibley (Washington university) : Brown, Kevin M et al. “Plasma Membrane Association by N-Acylation Governs PKG Function in Toxoplasma gondii.” mBio vol. 8,3 e00375-17. 2 May. 2017, doi:10.1128/mBio.00375-1. SAS6L-mAiD-3HA (HXGPRT) and DCX-mAiD-3HA have been generated by transfecting a Cas9-gRNA encoding vector alongside a PCR fragment encoding for mAiD-HA and HXGPRT cassette (allowing the selection of positive parasites by addition of Mycophenolic acid and Xanthine in the media).

Authentication

U2OS cell line was authenticated by profiling using polymorphic short tandem repeat loci (STR) (Microsynth). The other cell lines were not authenticated.

Mycoplasma contamination

Cells were regularly tested for mycoplasma contamination and were not positive for mycoplasma

Commonly misidentified lines
(See ICLAC register)

no commonly misidentified cell lines were used

Animals and other research organisms

Policy information about studies involving animals; ARRIVE guidelines recommended for reporting animal research, and Sex and Gender in Research

Laboratory animals	C57BL/6J mouse older than 2 month were used to obtain retinal tissue. Note that the sex was not determined.
Wild animals	no wild animals were used in this study
Reporting on sex	The sex was not determined
Field-collected samples	no filed-collected samples were used in this study
Ethics oversight	The retinas were dissected in the laboratory of Corinne Kostic and Yvan Arsenijevicm Hôpital ophtalmique Jules-Gonin, Lausanne, under the authorization VD1367. All ethics relative to work with animals were fully respected and experiments were carried our in accordance with the Institutional Animal Care of the Institute and Canton de Vaud.

Note that full information on the approval of the study protocol must also be provided in the manuscript.
